# Self-regulation facets differentially predict internalizing symptom trajectories from middle childhood to early adolescence: a longitudinal multimethod study

**DOI:** 10.1186/s13034-023-00670-3

**Published:** 2023-10-17

**Authors:** Johanna L. Klinge, Petra Warschburger, Robert Busching, Annette M. Klein

**Affiliations:** 1 International Psychoanalytic University Berlin, Stromstr. 1, 10555 Berlin, Germany; 2 Department of Psychology, University of Potsdam, Karl-Liebknecht-Str. 24-25, 14476 Potsdam, Germany

**Keywords:** Self-regulation, Internalizing symptoms, Trajectories, Childhood, Adolescence

## Abstract

**Background:**

Internalizing symptoms are among the most common psychological symptoms in childhood and adolescence, are highly stable and can cause severe impairment. Current research discusses lower capacities of self-regulation (SR) as risk factors for the development of internalizing symptoms. The present study identifies trajectories of internalizing symptoms in the transition phase from middle childhood to adolescence and examines multiple SR facets as predictors of potentially unfavorable trajectories, also in the presence of other established risk factors.

**Methods:**

The study utilized a community sample of *N* = 1453 (52.2% female) German children, who provided data at up to three measurement points (t1: 6–11 years, t2: 7–11 years, t3: 9–13 years). Trajectories of internalizing symptoms were based on parents’ ratings of the emotional problems scale of the Strengths and Difficulties Questionnaire. SR facets were assessed using multiple methods and informants. Two multinomial regression analyses were conducted to predict class membership by (1) SR facets and gender and (2) SR facets, gender, and other established risk factors (education status, family adversity, peer problems).

**Results:**

Using growth mixture modelling, we identified three trajectory classes with stable low (*n* = 1200), increasing (*n* = 124), and early high decreasing internalizing symptoms (*n* = 129). In the regression analysis controlling for risk factors, membership in the increasing trajectory was significantly predicted by higher emotional reactivity (*OR* = 2.65, *p* < .001), higher cognitive flexibility/set-shifting (*OR* = 1.48, *p* = .032), and higher family adversity (*OR* = 1.38, *p* = .046). Membership in the early high decreasing trajectory was significantly predicted by higher emotional reactivity (*OR* = 4.15, *p* < .001), higher inhibitory control (*OR* = 1.47, *p* = .045), lower working-memory updating (*OR* = 0.69, *p* = .016), lower delay of gratification (*OR* = 0.75, *p* = .028), and higher family adversity (*OR* = 1.63, *p* = .001).

**Conclusions:**

SR facets incrementally and differentially predict potentially unfavorable trajectories of internalizing symptoms from age 6 to 13, surpassing the predictive value of gender or education status. Higher emotional reactivity emerged as the most influential factor, which could therefore be addressed in future prevention and intervention efforts.

**Supplementary Information:**

The online version contains supplementary material available at 10.1186/s13034-023-00670-3.

## Introduction

Middle childhood and early adolescence encompass the developmental period from about age 6 to 13 and are characterized by significant cognitive, emotional, and social changes. During this critical phase, children’s internalizing symptoms, including anxiety, depression, somatic symptoms and withdrawal [1], can manifest and impact children’s well-being, family and school environment [2]. With prevalence rates of 11.2% of German 7- to 19-year-olds suffering from clinically relevant depressive symptoms and 10.6% of the same age group showing clinically relevant anxiety symptoms [3], internalizing symptoms are among the most common mental health problems at this age. However, the onset and the stability of these symptoms can vary across individuals.

### Developmental trajectories of internalizing symptoms

Following a developmental psychopathology framework [4], studies using group-based trajectory modelling have burgeoned in the last two decades, allowing for a more heterogeneous, person-centered specification of differential trajectories of symptoms. In these studies, the number of identified trajectories of internalizing symptoms varies between three and six, which differ in severity (low, medium, high) and stability (stable, increasing/decreasing) of symptoms [e.g., 5, 6, 7, 8, 9]. Most participants in these studies reported stable low or no symptoms, while a minority (usually < 10%) suffered from stable high symptoms [10]. Given the high risk of chronification in adulthood [11], and to devise targeted strategies to promote healthy socio-emotional development in children, it is critical to identify predictors of unfavorable trajectories of internalizing symptoms, such as increasing or stable high symptoms.

### Self-regulation facets as potential predictors

In recent years, the role of various facets of self-regulation (SR) as potential predictors of internalizing symptoms has become a focus of research. In general, SR can be defined as the ability to control and modulate one’s cognitions, behaviors, emotions, and physiological responses in order to attain future benefits [12–14]. However, as a multidimensional construct, SR serves as an umbrella term for many different SR facets, which are sometimes used inconsistently [for a comprehensive overview see 13]. Most studies linking SR to internalizing symptoms focus on single or only few SR facets [15, 16], whereas little research has examined various SR facets simultaneously as predictors of unfavorable trajectories of internalizing symptoms and thus can compare their predictive value. To address this research gap, the current study examines multiple SR facets which include working-memory updating, cognitive flexibility/set-shifting, inhibition, inhibitory control, planning behavior, affective decision-making, delay of gratification, emotional reactivity, anger regulation, and heart-rate variability. Additionally, several established risk factors (e.g., female gender) are considered to test the incremental contribution of SR facets in predicting unfavorable trajectories of internalizing symptoms.

#### Executive functions

Multiple SR facets examined in this study are known as executive functions (EF), for example the three core EF [17, 18]: working-memory updating (i.e., the ability to mentally hold and manipulate information), cognitive flexibility/set-shifting (i.e., the capacity to switch between different tasks, perspectives, or rules), and inhibition or inhibitory control (i.e., the ability to suppress inappropriate responses and inhibit impulsive behaviors). These can be considered as basal cognitive facets of SR and play a vital role in adjusting to new situational demands and social contexts. From these, more complex EF such as planning behavior (i.e., the ability to set goals, formulate strategies, and organize actions accordingly) develop [18, 19]. Based on considerable behavioral and neural evidence, these cognitive abilities are often referred to as “cool” EF as opposed to “hot” EF, which refer to emotion-driven processes [20]. Hot EF include affective decision-making (i.e., the process of integrating emotions and cognitions when making choices), and delay of gratification (i.e., the ability to postpone immediate rewards for future benefits).

The influence of cool and hot EF on the development of internalizing symptoms was recently evaluated in a large meta-analysis of 167 longitudinal studies during childhood and adolescence [16]. The authors reported that lower capacities of EF during childhood were associated with higher levels of later internalizing symptoms (*k* = 178, *r* = −.07). However, different correlations were found depending on the types of EF and of symptoms: Only cool EF were significantly negatively associated with later internalizing symptoms (*r* = −.07), while hot EF were not [16]. As only few studies exist on hot EF, this result can be considered preliminary. Moreover, EF were only significantly negatively associated with later depressive symptoms (*k* = 88, *r* = −.05) but not with later anxiety symptoms [16]. One study even found associations between higher capacities of EF and higher symptom burden [21]. These remaining inconsistencies demand further examination of the differential impact of single EF on developmental trajectories of internalizing symptoms.

#### Emotional reactivity and emotion regulation

Further SR facets that have been consistently associated with internalizing symptoms are emotional reactivity and emotion regulation. Emotional reactivity is considered a basal temperament aspect of SR determining the emotional reaction to events in terms of response threshold, latency, amplitude, rise time to peak intensity, and recovery time [22]. Adolescents who reported higher emotional reactivity showed higher depressive symptoms [23, 24], physical anxiety symptoms, and social anxiety symptoms [23]. Additionally, higher negative affectivity (i.e., a higher tendency to experience negative moods including worry, sadness and anger), which is a closely related construct, correlated positively with internalizing symptoms in both childhood and adolescence [25]. High negative affectivity also predicted the membership in increasing [e.g., 5, 6] or stable high [26] trajectories of internalizing symptoms demonstrating its critical value in early detection of unfavorable trajectories of internalizing symptoms.

Emotion regulation is a complex SR facet, which is considered to involve many of the abilities mentioned previously, and refers to “processes used to manage and change if, when, and how (e.g., how intensely) one experiences emotions and emotion-related motivational and physiological states, as well as how emotions are expressed behaviorally” [27 p.288]. In a meta-analysis of 35 studies during adolescence, adaptive emotion regulation strategies were negatively (*k* = 4–10, *r* = −.30 – −.46), whereas maladaptive emotion regulation strategies were positively associated with anxiety and depression symptoms (*k* = 6–25, *r *= .22 – .51) [28]. Regarding internalizing symptoms, another meta-analysis of 212 studies during childhood and adolescence also confirmed that these were negatively associated with better emotion regulation in cross-sectional studies (*k* = 15, *r* = −.23) [29]. However, this association was not found in longitudinal studies. The authors attribute this to the lack of longitudinal studies, but also consider it possible that poorer emotion regulation is causal only in individuals with more severe symptoms and not in those with less severe symptoms [29]. This underscores the importance of examining heterogeneous trajectories of internalizing symptoms and their differential predictors.

#### Heart rate variability

As a physiological SR facet, heart rate variability (HRV) reflects the variation of intervals between heartbeats. Higher HRV is considered a sign of better physiological adaptation to emotional stimuli and therefore more efficient SR [30]. Accordingly, abnormally low resting HRV has been found to be associated with higher internalizing [31, 32], anxiety [33], and depressive symptoms [34].

### Further established risk factors

As mentioned previously, this study additionally includes established risk factors to test the incremental contribution of SR facets in predicting unfavorable trajectories of internalizing symptoms. According to numerous studies, important risk factors for internalizing symptoms are female gender (gender differences in depressive symptoms occur after the onset of puberty) [35], lower education of parents [10], problems in peer relationships [10, 36], and higher family adversity [10, 37], which is also associated with higher psychiatric symptoms and disorders in general [38].

### Current study and hypotheses

There are major research gaps regarding the contribution of SR facets to the development of internalizing symptoms. Firstly, there is a lack of longitudinal studies examining SR facets as predictors of trajectories of internalizing symptoms during the transition from middle childhood to early adolescence. However, since associations between SR facets and internalizing symptoms may differ depending on symptom level [29], understanding how SR facets relate to different trajectories of internalizing symptoms is crucial. Secondly, most studies examine only one or a few SR facets neglecting the simultaneous examination of multiple SR facets and their incremental contribution beyond established risk factors. Thus, they cannot determine which SR facets and risk factors are most influential.

To address these research gaps, we conducted a prospective study assessing internalizing symptoms at three measurement points from middle childhood to early adolescence. Our objectives were to identify trajectories of internalizing symptoms, examine the differential predictive contributions of SR facets to the membership in unfavorable trajectories, and determine whether SR facets incrementally contribute to this prediction also when established risk factors are included. Based on previous research, we expected to identify at least three trajectories of internalizing symptoms, with at least two of these showing stable courses and at least one of these showing increasing symptoms. Furthermore, we expected membership in unfavorable trajectories to be negatively associated with all SR facets, except for emotional reactivity which we expected to be positively associated. With respect to the established risk factors, we expected family adversity and peer problems to be positively associated, and education status to be negatively associated with internalizing symptoms. We explored gender differences without specific predictions, given the age range of our sample.

The study’s hypotheses and analysis plan were preregistered to ensure transparency and reduce publication bias, see 10.17605/OSF.IO/RQM5N. Minor deviations from preregistration are explained in supplementary Table S1 [see Additional file 1].

## Methods

### Sample and procedure

The data of this study were collected as part of a large longitudinal study on intrapersonal developmental risk factors in childhood and adolescence conducted at the University of Potsdam, Germany (for an overview, see study protocol [39]). Participants provided data at up to three measurement points (t1–t3) in childhood and early adolescence. Participating families were recruited from 120 classes in 33 public primary schools in the Federal State of Brandenburg, Germany. Assessments were self-, parent-, and teacher-rated. At t1, 1657 children (52.2% girls), aged between 6 and 11 years (*M*_*age*_ = 8.36, *SD* = 0.95), and 1340 (80.7%) parents participated. At t2, 1612 children (51.9% girls; *M*_*age*_ = 9.11 years, *SD* = 0.93; 97.5% retention rate) and 1197 (72.1%) parents took part on average 9.14 months (*SD* = 1.80) after t1. At t3, 1534 children (51.7% girls, *M*_age_ = 11.06 years, *SD* = 0.92; 92.6% retention rate) and 1070 (64.5%) parents participated again, on average 23.83 months (*SD* = 1.66) after t2. Parent-questionnaires were completed by mothers (69–77%), by both parents jointly (12.1–20.4%), by fathers (6.8–7.2%) or by other caregivers (0.8−1.5%).

In this study, we examined the developmental trajectories of internalizing symptoms based on parent-reported data collected at t1, t2, and t3. To account for missing data, we employed full information maximum likelihood (FIML) estimation as proposed by Little and Rubin [40]. This approach allowed us to include all children whose parents reported their symptoms at least once, resulting in a final sample size of *N* = 1453. Table 1 provides an overview of the sociodemographic characteristics of the final sample and their respective families. Importantly, the final sample (*N* = 1453) did not differ significantly from the total sample of participants at t1 (*N* = 1657) in terms of age, gender, domestic situation, or parents’ highest education degree (all *p*s > .920). Predictors, including SR facets and risk factors, were assessed at t1, except for family adversity, which was only available at t2.

**Table 1:** Sociodemographic characteristics of the final sample (*N* = 1453)

	*n*	(%)
Females (measured at t1)	758	(52.2%)
Domestic situation (measured at t1)		
Lives with both parents	971	(66.8%)
Lives with one parent and new partner	84	(5.8%)
Lives with only one parent	21	(1.5%)
Another social situation	9	(0.6%)
Missing	368	(25.3%)
Household income (measured at t2)		
less than €2000/month	218	(15.0%)
€2000 to €5000/month	729	(50.2%)
more than €5000/month	178	(12.3%)
Missing	328	(22.6%)
Highest education degree of both parents (measured at t1)		
No degree / Special school	14	(0.9%)
Lower secondary school	34	(2.3%)
Upper secondary school	418	(28.8%)
High school diploma	257	(17.7%)
University degree	602	(41.4%)
Missing	128	(8.8%)

Participation in the study was voluntary. Parents and children provided informed consent after receiving information about the study. All assessments were approved by the Research Ethics Board at the University of Potsdam and by the Ministry of Education, Youth, and Sport of the Federal State of Brandenburg, Germany. The children completed a standardized test battery in two testing sessions each lasting one hour. The participants obtained small gifts and cinema vouchers for their participation. Parents and teachers filled in questionnaires either online or in printed format. Teachers received 5€ for the class fund for each child for whom they filled in questionnaires.

### Measures

Our main outcome measure, internalizing symptoms, was assessed with the emotional problems scale of the Strengths and Difficulties Questionnaire (SDQ) [41] at t1, t2, and t3. The scale contains 5 items about anxiety, depressive, and psychosomatic symptoms (e.g., “Many fears, easily scared”), scored by parents as *not true* (0), *somewhat true* (1), or *certainly true* (2) in the last 6 months (sum score 0–10). With respect to German norm data, sum scores of 0 to 3 are classified as “close to average”, scores of 4 as “slightly raised”, scores of 5 to 6 as “high” and scores of 7 to 10 as “very high” [42]. Adequate reliabilities were confirmed at all measurement points (α = .66 − .72).

#### SR facets

Using a multimethod approach, SR facets were assessed at t1 with a variety of questionnaires completed by different informants, computer-based experiments, and a physiological measurement.

Working-memory updating was measured experimentally by the sum of correctly answered sequences (max. 16) in the digit-span backwards task (ZN-R) from the German version of the Wechsler Intelligence Scale for Children – Fourth Edition (WISC-IV) [43]. Children were asked to repeat digit-spans backwards while the span length increased from 2 to 8 in a maximum of 8 trials. Each trial consisted of two sequences of the same length, and the test was stopped when a child incorrectly repeated both sequences during one trial.

Cognitive flexibility/set-shifting was measured experimentally by the number of correct responses in switch trials of the Cognitive Flexibility Task [44]. In 46 trials, children were asked to alternately feed plain and multi-colored fish with one type of fish displayed on the left side of the screen and another type on the right at the same time. In 22 randomly occurring switch trials, the plain and multi-colored fish switched sides, so response patterns had to be adapted.

Inhibition was measured experimentally by the interference score of the Fruit-Stroop task [45], an adapted version of the Stroop task [46]. Children were presented with four pages of 25 stimuli each under different conditions (pages 1 and 2: no interference, pages 3 and 4: interference). An interference score was calculated with a formula based on the time (in seconds) the children needed to name the colors of all 25 stimuli per page [time page 4 – (time page 1 x time page 3) / (time page 1 + time page 3)] [45]. The interference score was z-standardized and inverted so that higher values indicate higher inhibition capability.

Inhibitory control was measured by the mean score of 6 items of the inhibitory control scale of the Temperament in Middle Childhood Questionnaire (TMCQ) [47], rated by parents from *not at all* (1) to *a lot* (5). Higher values indicate higher inhibitory control and items showed an adequate reliability of α = .68.

Planning behavior was measured by the mean score of 8 items of the planning/organizing scale of the Behavior Rating Inventory of Executive Function (BRIEF) [48], rated by teachers on an adapted 5-point scale ranging from *never* (1) to *always* (5). Items were reverse scored so that higher values indicate higher planning behavior. Items showed a high reliability of α = .93.

Affective decision-making was measured experimentally by the net-score difference between advantageous and disadvantageous choices in the Hungry Donkey Task [49], a computer based, age-appropriate version of the Iowa Gambling Task [50]. The task encompassed 10 practice and 50 test trials in which children could choose between four doors with different win/lose contingencies.

Delay of gratification was measured experimentally by the sum of delayed rewards in 4 questions in which children were asked whether they wanted to obtain smaller rewards (2 sweets, 2 toys) instantly or larger rewards later.

Emotional reactivity was measured by the mean score of 10 items of the emotional control scale of the BRIEF [48], rated by parents on an adapted 5-point scale from *never* (1) to *always* (5). Higher values indicate higher emotional reactivity, and items showed a high reliability of α = .91.

Anger regulation was measured by the mean score of 4 items of the dealing with rage scale of the German Questionnaire on Emotion Regulation in Children and Adolescents (FEEL-KJ) [51], rated by parents from *never* (1) to *often* (3). Following the procedure of Rohlf and Krahé [52], two items assessing the anger regulation strategies perseveration and venting were recoded after being classified as maladaptive strategies as opposed to the other two items assessing the strategy distraction, which was classified as an adaptive strategy. Items showed an acceptable reliability of α = .59.

HRV was measured by the root mean square of successive differences (RMSSD; in ms) during a three-minute resting phase, assessed by using a strap with two electrodes, which was fixed to the table and attached to the children’s hands. The electrodes were connected to the polar watch RS800CX (Polar Electro Oy, Kempele, Finland), a mobile device enabling non-invasive recording of heartbeat intervals. Signals were sampled at 1000 Hz and analyzed with the corresponding Polar ProTrainer 5 software (version 5.40.172) [53]. Higher values indicate higher variability.

#### Risk factors

Children rated their gender as *female* (1) or *male* (2), and parents specified their highest education degree from *no school degree* (1) to *university degree* (6), which served as an indicator of the education status of the household.

Family adversity was measured by a sum score of existing family risk factors that children had experienced until t2, based on 9 items (e.g., mental illness of a parent, separation or divorce of parents, or frequent parental quarrels), which were adapted from the family adversity index by Rutter and Quinton [54] and the German translated version by Voll and Allehoff [55], and were rated by parents as *not present* (0) or *present* (1).

Peer problems were assessed with the eponymous scale of the SDQ [41], rated by parents (5 items) and teachers (3 items) from *not true* (0) to *certainly true* (2). The internal consistency was α = .65 for the parents’ scale and α = .70 for the teachers’ scale. To take into account both informants, a mean score was calculated from the parents’ and teachers’ mean scores, resulting in an internal consistency of α = .52. Given the fact that this scale contains only these two items reflecting different environmental contexts (home and school), this reliability is considered adequate.

### Statistical analyses

We estimated growth mixture models of internalizing symptoms with the maximum likelihood estimator with robust standard errors (MLR) using Mplus (version 8.8) [56]. To address missing data, we utilized full information maximum likelihood (FIML) estimation [40]. Considering the variation in children’s ages across the three measurement points (e.g., ages 6 to 11 years at t1), we specified individually varying times of observation using the TSCORES function [56]. This allowed us to encompass the age range from 6 to 13 years in our analyses.

We conducted a series of models with increasing numbers of classes, commencing with a general growth model consisting of a single class. To determine the best-fitting model, we compared various fit indices, including the Akaike information criterion (AIC), Bayesian information criterion (BIC), and sample-size adjusted Bayesian information criterion (aBIC). Lower values of these fit indices indicate a better fit to the data. Additionally, we calculated entropy, a measure of class distinctness and classification accuracy ranging from zero to one. Higher entropy values indicate greater separation between classes and better categorization of participants within those classes [57].

After identifying the best fitting solution, we performed two multinomial logistic regression analyses. The first regression analysis included z-standardized scores of the 10 facets of self-regulation (SR) and gender as independent variables. In the second regression analysis z-standardized scores of further established risk factors for internalizing symptoms (educational status, family adversity, peer problems) were added as independent variables. In both analyses, class membership served as the dependent variable, with the largest and most resilient class serving as the reference group.

## Results

First, the basic developmental course of internalizing symptoms was estimated by a general growth model. This resulted in a mean intercept (mean level of internalizing symptoms at age 6) of 1.68, which differed significantly from zero (*p* < .001) and a slope of 0.008, which did not significantly differ from zero (*p* = .701). Considering SDQ norm data, the sample on average showed a “close to average” (SDQ cut-off = 0–3) symptom level. The interindividual variance was significant in both the intercept (σ^2^ = 2.52, *p* < .001) and the slope (σ^2^ = 0.08, *p* = .032), which indicates that there were participants with different symptom levels at age 6 as well as changing symptom levels over time. Hence, deriving different trajectory classes for internalizing symptoms was justified.

### Estimation of latent trajectory classes

With increasing number of classes, the fit indices AIC, BIC, and aBIC consistently improved (see Table 2). However, this was accompanied by a decrease in class sizes, rendering further analyses unfeasible (e.g., one class with only *n* = 8 in the seven-class solution), which suggests over-parametrization. Considering previous research [6, 58] and aiming for parsimony, we opted for the three-class solution consisting of a “stable low”, an “increasing”, and an “early high decreasing” class. These classes demonstrated a good entropy of 0.81. Comparisons of the three-class solution with higher-class solutions revealed that the additional classes did not provide theoretically meaningful information to differentiate between participants. For example, in the four-class solution, the “early high decreasing” class from the three-class solution was split into two “decreasing” classes with slightly different intercepts, and in the five-class solution, the “increasing” class from the three-class solution was further divided into two “increasing” classes with slightly different intercepts.

**Table 2:** Model fit statistics for growth mixture models with increasing number of trajectory classes

	AIC	BIC	aBIC	Entropy	Smallest *n*
1 class	13289.11	13331.36	13305.95		1453
2 classes	13009.12	13067.21	13032.27	0.82	255
3 classes	12842.10	12916.04	12871.57	0.81	124
4 classes	12770.70	12860.49	12806.48	0.77	59
5 classes	12725.53	12831.16	12767.63	0.79	24
6 classes	12672.87	12794.34	12721.28	0.72	30
7 classes	12627.05	12764.36	12681.49	0.77	8

*Note*: For AIC (Akaike information criterion), BIC (Bayesian information criterion), and aBIC (sample-size adjusted BIC) lower values indicate better fitting models. For entropy higher values indicate better quality of class assignment. Smallest *n* refers to the class with the smallest number of participants.

**Fig. 1:**
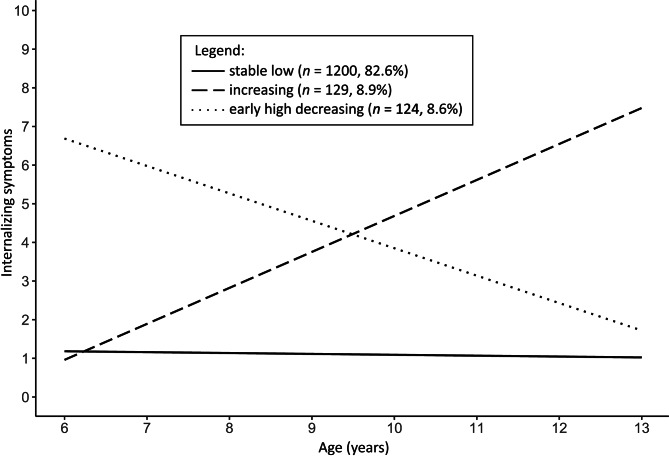
Estimated trajectories of internalizing symptoms from age 6 to 13

The three estimated trajectories of internalizing symptoms between ages 6 and 13 are illustrated in Fig. 1. The largest group of children belonged to a “stable low” class (*n* = 1200, 82.6%, 615 females) with “close to average” (SDQ cut-off = 0–3) initial levels of internalizing symptoms (*b* = 1.18, *p* < .001) and no change over time (*b* = -0.02, *p* = .502). 129 children (8.9%, 74 females) belonged to an “increasing” class with “close to average” initial levels of internalizing symptoms (*b* = 0.96, *p* = .013) that increased as they grew older (*b* = 0.93, *p* < .001) to “high” (SDQ cut-off = 5–6) levels. 124 children (8.5%, 69 females) were assigned to an “early high decreasing” class with “high” initial levels of internalizing symptoms (*b* = 6.69, *p* < .001) that decreased over time (*b* = -0.71, *p* < .001) to levels “close to average”. The posterior probability of being assigned to the right class (ranging from zero to one) was .81 for the “stable low” class, .77 for the “increasing” class, and .95 for the “early high decreasing” class.

#### Preliminary analyses

Classes did not differ with respect to gender, 𝜒^2^ (2, *N* = 1453) = 2.40, *p* = .298, and age at T1, *F* (2, *N* = 1450) = 2.55, *p* = .078.

Bivariate correlations of all variables examined are presented in supplementary Table S2 [see Additional file 2], and descriptive data of SR facets and risk factors for the latent trajectory classes are presented in supplementary Table S3 [see Additional file 3].

### Prediction of class membership by SR facets and other risk factors

After identifying the three-class solution, we performed two multinomial logistic regression analyses, the first with SR facets and gender and the second additionally with further established risk factors as predictors for class membership. The individual parameter estimates of both regression analyses can be found in Table 3.

**Table 3:** Parameter estimates from two multinomial logistic regression analyses, both with class membership as the dependent variable, while in the first SR facets and gender, and in the second additionally added risk factors form the independent variables

	First regression analysis					Second regression analysis			
		95% CI for odds ratios					95% CI for odds ratios		
	*B* (*SE*)	Lower	Odds ratio	Upper		*B* (SE)	Lower	Odds ratio	Upper
*Class 2 (increasing) vs. class 1 (stable low)*									
Working-memory updating	-0.28 (0.16)^+^	0.56	0.75	1.03		-0.30 (0.16)^+^	0.54	0.74	1.00
Cognitive flexibility/set-shifting	0.29 (0.17)^+^	0.96	1.33	1.85		0.39 (0.18)*	1.04	1.48	2.11
Inhibition	0.10 (0.16)	0.80	1.10	1.50		0.05 (0.17)	0.76	1.05	1.45
Inhibitory control	0.02 (0.19)	0.70	1.02	1.47		0.17 (0.21)	0.79	1.18	1.77
Planning behavior	-0.50 (0.17)**	0.43	0.60	0.85		-0.27 (0.19)	0.52	0.76	1.11
Affective decision-making	-0.19 (0.17)	0.60	0.83	1.15		-0.16 (0.16)	0.61	0.86	1.21
Delay of gratification	-0.21 (0.14)	0.61	0.81	1.07		-0.23 (0.16)	0.59	0.80	1.08
Emotional reactivity	1.03 (0.21)***	1.85	2.81	4.27		0.97 (0.23)***	1.70	2.65	4.13
Anger regulation	-0.18 (0.30)	0.46	0.83	1.49		-0.21 (0.29)	0.46	0.81	1.42
Heart-rate variability	-0.11 (0.17)	0.65	0.90	1.25		-0.16 (0.17)	0.61	0.85	1.19
Gender	-0.43 (0.32)	0.35	0.65	1.22		-0.29 (0.33)	0.39	0.75	1.42
Education status						-0.27 (0.17)^+^	0.55	0.76	1.05
Family adversity						0.32 (0.16)*	1.01	1.38	1.89
Peer problems						0.35 (0.19)^+^	0.99	1.42	2.04
*Class 3 (early high decreasing) vs. class 1 (stable low)*									
Working-memory updating	-0.37 (0.17)*	0.50	0.69	0.96		-0.37 (0.15)*	0.52	0.69	0.93
Cognitive flexibility/set-shifting	0.15 (0.16)	0.85	1.17	1.60		0.23 (0.17)	0.90	1.25	1.74
Inhibition	0.18 (0.15)	0.89	1.20	1.62		0.12 (0.14)	0.85	1.13	1.49
Inhibitory control	0.31 (0.20)	0.92	1.37	2.02		0.38 (0.19)*	1.01	1.47	2.13
Planning behavior	-0.55 (0.17)**	0.41	0.58	0.80		-0.32 (0.20)	0.50	0.73	1.07
Affective decision-making	0.23 (0.15)	0.94	1.26	1.70		0.26 (0.15)^+^	0.97	1.30	1.74
Delay of gratification	-0.25 (0.13)*	0.61	0.78	1.00		-0.29 (0.13)*	0.58	0.75	0.97
Emotional reactivity	1.50 (0.23)***	2.87	4.50	7.05		1.43 (0.23)***	2.65	4.15	6.50
Anger regulation	0.16 (0.19)	0.81	1.18	1.72		0.09 (0.19)	0.74	1.09	1.59
Heart-rate variability	0.07 (0.16)	0.78	1.07	1.48		0.05 (0.17)	0.76	1.06	1.47
Gender	-0.29 (0.30)	0.42	0.75	1.36		-0.18 (0.31)	0.45	0.83	1.53
Education status						-0.14 (0.17)	0.63	0.87	1.20
Family adversity						0.49 (0.14)**	1.23	1.63	2.16
Peer problems						0.24 (0.16)	0.93	1.27	1.74

^+^*p* < .10, *p < .05, **p < .01, ***p < .001

#### Predictors for distinguishing between the increasing and stable low classes

The first regression analysis showed that lower planning behavior (*OR* = 0.60, *p* = .003) and higher emotional reactivity (*OR* = 2.81, *p* < .001) were significantly associated with a heightened probability of belonging to the increasing rather than the stable low class. After including established risk factors in the second regression analysis, only emotional reactivity (*OR* = 2.65, *p* < .001) remained significant and higher cognitive flexibility/set-shifting (*OR* = 1.48, *p* = .032) became significant. Only one added risk factor, higher family adversity (*OR* = 1.38, *p* = .046), reached significance.

#### Predictors for distinguishing between the early high decreasing and stable low classes

The first regression analysis showed that lower working-memory updating (*OR* = 0.69, *p* = .029), lower planning behavior (*OR* = 0.58, *p* = .001), lower delay of gratification (*OR* = 0.78, *p* = .047), and higher emotional reactivity (*OR* = 4.50, *p* < .001) were significantly associated with a heightened probability of belonging to the early high decreasing rather than the stable low class. After including established risk factors in the second regression analysis, working-memory updating (*OR* = 0.69, *p* = .016), delay of gratification (*OR* = 0.75, *p* = .028), and emotional reactivity (*OR* = 4.15, *p* < .001), but not planning behavior, remained significant. Instead, higher inhibitory control (*OR* = 1.47, *p* = .045) and one added risk factor, higher family adversity (*OR* = 1.63, *p* = .001), became significant.

## Discussion

In this large community-based longitudinal study, we identified three distinct trajectories characterizing the development of internalizing symptoms during the transition from middle childhood to early adolescence: a stable low class, an increasing class, and an early high decreasing class. In contrast to many prior studies that focused on examining individual or limited SR facets as predictors, we simultaneously investigated multiple SR facets using a multimethod approach. Further, we investigated their incremental contribution in predicting the increasing and early high decreasing trajectories of internalizing symptoms, while considering the presence of other well-established risk factors. Before delving into the specific results for each trajectory, it is crucial to emphasize that among the included risk factors, only higher family adversity was associated with an increased likelihood of belonging to both aforementioned trajectories. This underscores the clear significance of the SR facets in predicting internalizing symptoms, surpassing the relevance of other factors such as gender, educational status, or peer problems.

### Trajectories of internalizing symptoms

The trajectories identified in our study are largely consistent with our hypotheses, with the exception that we expected to find at least two stable trajectories, as suggested by the broad evidence for stable high trajectories [e.g., 8, 58, 59] besides stable low trajectories. However, this deviation can likely be attributed to our community-based sample, as other studies using similar samples have also failed to identify a stable high trajectory [6, 60]. Consistent with previous findings [e.g., 7, 8, 10, 61], the largest proportion of children (82.6%) belonged to the stable low trajectory, while the remaining participants were equally assigned to the increasing and early high decreasing trajectories. According to the SDQ norm data [42], children in the increasing trajectory exhibited initial symptoms close to the average, which subsequently escalated to high symptom levels over time. In contrast, the early high decreasing trajectory initially exhibited high symptoms on average, which then gradually decreased. While the increasing trajectory clearly represents an unfavorable course, it remains uncertain whether the same can be concluded about the early high decreasing trajectory. Participants in the decreasing trajectory descriptively exhibited higher symptoms compared to those in the stable low class across the investigated age range, suggesting a lingering risk of redeveloping higher internalizing symptoms in the future. The onset of puberty, particularly for girls, is associated with heightened vulnerability to internalizing [62] and depressive symptoms [35]. Furthermore, anxiety symptoms tend to decrease during early adolescence and then rise again during middle or late adolescence [63]. In line with this, Sterba et al. [8] identified a trajectory where symptoms initially decreased and then increased again. However, since in our study statistical analyses revealed no significant differences between all three trajectories in terms of age or gender, it is unlikely that these variables would explain the different trajectory patterns. Instead, it is plausible that participants in the early high decreasing trajectory, or their families, have developed better coping mechanisms helping to reduce the children’s early high symptoms. Consequently, we consider the increasing trajectory an unfavorable course while children following an early high decreasing course could show a remaining risk. Accordingly, it is crucial to gain a comprehensive understanding of the similarities and differences between children following a stable low trajectory which can be considered the most favorable course and children following other developmental pathways.

### SR facets as predictors for the increasing trajectory of internalizing symptoms

Higher emotional reactivity and higher cognitive flexibility/set-shifting were associated with an increased likelihood of belonging to the increasing trajectory compared to the stable low trajectory, even when established risk factors were taken into account. However, in the first regression analysis without risk factors, cognitive flexibility/set-shifting was not significant, but planning behavior was, as further discussed below. Interestingly, emotional reactivity was significant in both regression analyses, displaying notably higher odds ratios than other predictors, indicating its significant influence. This finding aligns with our hypothesis, which was supported by a meta-analysis conducted by Kostyrka-Allchorne et al. [64], indicating a positive association between negative emotionality in early childhood and later development of internalizing symptoms. Similarly, two other studies reported that higher levels of negative affectivity predicted membership in increasing trajectories [e.g., 5, 6], while another study found that difficult temperament did not predict the onset of internalizing symptoms during adolescence [61]. It is noteworthy that the effect of emotional reactivity on the probability of membership in the increasing trajectory in our sample is substantial, surpassing the magnitude observed in the meta-analysis by Kostyrka-Allchorne et al. [64]. This suggests that heightened emotional reactivity may serve as an early observable marker and a highly promising target for preventive interventions in children who have not yet exhibited internalizing symptoms.

Regarding cognitive flexibility/set-shifting, our findings contradict our initial hypothesis and current meta-analyses [15, 16] demonstrating negative associations between cool EF and subsequent internalizing symptoms. Likewise, our results challenge the theoretical framework proposed by Henderson and colleagues [65, 66], wherein attention shifting is regarded as a protective factor against specific anxiety symptoms through the enhancement of dynamic social information processing. Different explanations for this can be considered. For example, higher cognitive flexibility/set-shifting might amplify the impact of other risk factors, ultimately leading to an escalation in internalizing symptoms. It is also possible that both increased cognitive flexibility/set-shifting and internalizing symptoms stem from a common cause, with the former manifesting earlier. For example, a study conducted by Mittal et al. [67] revealed that adults who had experienced an unpredictable childhood demonstrated greater proficiency in set-shifting, as adaptability is particularly advantageous in unpredictable environments. However, it is worth considering the potential occurrence of a suppressor effect, as cognitive flexibility/set-shifting exhibited significance solely in the regression analysis with and not the one without established risk factors. This limitation warrants cautious interpretation and underscores the need for further longitudinal investigations.

Planning behavior was significantly negatively associated with membership in the increasing trajectory, but only in the first regression analysis without established risk factors. This suggests that, in the presence of other risk factors, lower planning capabilities may not be of relevance. Nonetheless, given that teachers can readily observe and assess this predictor, as done in our study, lower planning behavior should still be regarded as an indicative marker for increasing symptoms within the school environment, if its relevance is further confirmed in future investigations.

### SR facets as predictors for the early high decreasing trajectory of internalizing symptoms

SR facets that demonstrated a significant association with an increased likelihood of belonging to the early high decreasing trajectory compared to the stable low trajectory, even in the presence of other established risk factors, encompassed higher emotional reactivity, lower working-memory updating, lower delay of gratification, and higher inhibitory control. Again, our findings regarding emotional reactivity align with our initial hypothesis and are consistent with the meta-analysis conducted by Kostyrka-Allchorne et al. [64]. Interestingly, the odds ratio for emotional reactivity is even higher for the early high decreasing trajectory compared to the increasing trajectory. Similar patterns, i.e., that odds ratios correspond to the initial symptom levels of trajectories, have been reported in several studies reviewed by Musliner et al. [10], leading them to suggest this as a dose-response pattern. Nevertheless, it is surprising that the group exhibiting the highest emotional reactivity demonstrates a decline in symptoms over time. It is plausible that individuals belonging to this trajectory, or significant persons in their environment, develop improved coping mechanisms to manage their heightened reactivity as time progresses.

In accordance with our hypothesis and two meta-analyses [15, 16], working-memory updating displayed a negative association with membership in the early high decreasing trajectory, as observed in regression analyses with and without established risk factors. However, it did not predict membership in the increasing trajectory. This suggests that working-memory updating may not be a significant contributing factor in the development of internalizing symptoms, but rather a consequence or byproduct of pre-existing symptoms, as indicated by reviews of case-control studies conducted by Baune et al. [68] and Vilgis et al. [69]. Furthermore, these reviews highlight that the impact of EF might vary depending on the severity of symptoms. Nonetheless, it is crucial to emphasize that working-memory updating consistently demonstrated significance in both regression analyses, with and without other risk factors, predicting the early high decreasing trajectory thus indicating its particular importance in this context.

Delay of gratification but not affective decision-making, as indicators of hot EF, showed a significant negative association with membership in the early high decreasing trajectory, partly supporting our hypothesis. Earlier findings were inconsistent, e.g. the meta-analysis by Yang et al. [16] did not demonstrate associations between impaired hot EF and later internalizing symptoms, while two studies reported negative associations between affective decision-making and internalizing symptoms, but only in boys [70, 71]. Notably, delay of gratification, along with emotional reactivity and working-memory updating, was significant in both regression analyses, with and without established risk factors. Therefore, further longitudinal studies should investigate the strength and significance of this relationship.

Contrary to our expectations and previous research, inhibitory control showed a positive association with membership in the early high decreasing trajectory. While most studies link deficits in EF to depressive symptoms [15, 16], some find enhanced EF in individuals with anxiety symptoms [21, 72, 73]. This unexpected finding may suggest that participants belonging to the early high decreasing trajectory experience primarily anxiety rather than depressive symptoms. However, our measures did not allow to test this hypothesis. Furthermore, parent-rated inhibitory control may overlap with behavioral inhibition (BI), a related construct associated with withdrawal and elevated negative reactivity [74–77]. Longitudinal studies indicate that BI increases risk for later anxiety [78, 79], but not all highly inhibited children develop internalizing disorders [80]. Our results align with this, as inhibitory control did not predict membership in the increasing trajectory. Regarding the prediction of the membership in the early high decreasing trajectory, inhibitory control was only significant in the regression analysis with established risk factors, indicating a possible suppressor effect. Thus, it should be interpreted with caution.

In a manner akin to the findings concerning the increasing trajectory, there was a noteworthy negative association between planning behavior and membership in the early high trajectory, although this relationship emerged solely in the regression analysis that did not include established risk factors. Thus, it can be inferred that planning behavior, while not the most prominent factor, still holds relevance within the school context and should not be disregarded.

### Strengths and limitations

The findings of this study should be interpreted in light of its strengths and limitations. Data were obtained from a large community-based sample of children and adolescents over four years. The use of an accelerated longitudinal design, accounting for varying ages at different measurement points, allowed us to cover the critical transition phase from middle childhood to early adolescence when many mental disorders tend to emerge [81]. While our results may be generalizable to other community samples, they may not apply to clinical samples due to the absence of a stable high trajectory.

By simultaneously investigating multiple SR facets, we were able to assess their incremental contribution in predicting differential trajectories of internalizing symptoms, while considering the effect of other well-established risk factors. Consequently, our study provides valuable insights for prevention and intervention efforts, particularly by highlighting the significance of higher emotional reactivity, diverse EF capabilities, and family adversity. These predictors can be identified as important risk factors for the development or maintenance of internalizing symptoms in middle childhood, and could be targeted by interventions, if further studies confirm their relevance.

Another strength is the use of multiple methods to assess SR facets, including validated scales and experiments, with input from different informants (self, parents, and teachers). This approach mitigated the risk of overestimating associations due to shared method or informant variance. However, latent modelling as suggested by Kraemer et al. [82] was not feasible due to limited availability of multiple measures or informants for individual SR facets, potentially introducing biases. Additionally, we could not differentiate between specific anxiety and depressive symptoms or explore their distinct developmental trajectories.

The assessment of internalizing symptoms in this study relied exclusively on parent reports, which tend to yield lower scores when compared to self-reports during adolescence [83]. However, all participants at T1 and the majority at T2 were younger than 11 years and therefore too young to apply the self-rating version of the SDQ. In addition, a meta-analysis has shown that in children, it is mainly external informants (such as teachers) who underestimate internalizing symptoms, whereas parents have a higher sensitivity to their child’s emotions [84]. However, even an underestimation of individual results should have little impact on our results since we mainly focus on the relationship between the different constructs. Nevertheless, future studies should consider also including the perspective of the children themselves to enable a combination of parent and self-ratings.

Moreover, our findings are generally consistent with the growing body of research identifying SR or specific SR facets as transdiagnostic markers of psychopathology [e.g., 85, 86, 87]. In the narrow sense, however, this applies specifically to emotional reactivity [25] and EF [e.g., 15, 19]. In contrast to previous findings, no evidence was found for the relevance of HRV [31] and emotion regulation [88, 89], though we only tested specific anger regulation. It should also be emphasized that, opposed to most studies identifying SR or SR facets as transdiagnostic factors, we also identified positive associations between specific EF and potentially adverse trajectories of internalizing symptoms. Further research is needed to explore the possibility that not only lower but also higher abilities in SR might contribute to potentially unfavorable courses of internalizing symptoms. In addition, cross-lagged modelling could provide insight into potential bidirectional influences between SR facets and internalizing symptoms over time.

## Conclusion

Overall, our study demonstrates that SR facets incrementally and differentially contribute to the prediction of potentially unfavorable trajectories of internalizing symptoms from age 6 to 13, surpassing the predictive value of other well-established risk factors such as gender, education status or peer problems. Already during middle childhood, SR facets can serve as observable markers of existing or increasing internalizing symptoms, with higher emotional reactivity being the most influential factor. Additionally, not only lower EF (working-memory updating, delay of gratification) but also higher EF (inhibitory control, cognitive flexibility/set-shifting) appear to contribute to the development or maintenance of internalizing symptoms. Our findings imply distinct relationships between specific SR facets and potentially unfavorable trajectories of internalizing symptoms, which should be addressed in future prevention and intervention measures.

### Electronic supplementary material

Below is the link to the electronic supplementary material.

Additional file 1: Table S1. Deviations from preregistration

Additional file 2: Table S2. Bivariate correlations of all examined variables

Additional file 3: Table S3. Descriptive data on SR facets and risk factors for the latent trajectory classes

## Data Availability

The datasets used and analyzed during the current study are not publicly available, as the participants were not asked to consent to publication within repositories but are available from the corresponding author on reasonable request.
