# Supplementary material for: Self-regulation facets differentially predict internalizing symptom trajectories from middle childhood to early adolescence: a longitudinal multimethod study
Source: Child Adolesc Psychiatry Ment Health. 2023 Oct 17;17:120. doi: 10.1186/s13034-023-00670-3 (PMC10583422; doi:10.1186/s13034-023-00670-3)
Supplement: Supplementary file 1 — Additional file 1: Table S1. Deviations from preregistration [file 13034_2023_670_MOESM1_ESM.docx]

**Table S1.** *Deviations from preregistration*

| **Deviation** | **Explanation** |
| --- | --- |
| Variable *behavioral control* was renamed to *inhibitory control.* | The variable was renamed to ensure uniform naming of the variables in the research group. Additionally, the variable’s new name corresponds with the name of the used subscale of the Temperament in Middle Childhood Questionnaire [TMCQ; 1]. |
| Variable *socioeconomic status* was renamed to *education status.* | Variable was renamed because it was measured by the highest education degree of both parents. This is supported by a meta-analysis by Musliner et al. [2] showing that lower education of parents is a risk factor for internalizing symptoms of children and adolescents. |
| Variable *peer acceptance* was renamed to *peer problems.* | The variable was renamed to correspond with the name of the used subscale of the Strengths and Difficulties Questionnaire [SDQ; 3]. |
| Variable *self-esteem* was excluded from analysis. | Variable was excluded because the scale showed inadequate internal consistency in our sample (α = .49). |
| In the latent class analysis, the best-fitting model was not determined by comparing different model fit-indices but by theoretical considerations and the criterion parsimony. | The latent class analysis led to over-parameterization. This made it necessary to change the procedure by which the best-fitting model was determined which is explained in detail in the results section of the manuscript. |

**References**

1. Simonds J, Kieras JE, Rueda MR, Rothbart MK. Effortful control, executive attention, and emotional regulation in 7–10-year-old children. Cogn Dev. 2007;22(4):474-88.
2. Musliner KL, Munk-Olsen T, Eaton WW, Zandi PP. Heterogeneity in long-term trajectories of depressive symptoms: Patterns, predictors and outcomes. J Affect Disord. 2016;192:199-211.
3. Goodman R. The Strengths and Difficulties Questionnaire: A Research Note. J Child Psychol Psychiatr. 1997;38(5):581-6.
