# Supplementary material for: Self-regulation facets differentially predict internalizing symptom trajectories from middle childhood to early adolescence: a longitudinal multimethod study
Source: Child Adolesc Psychiatry Ment Health. 2023 Oct 17;17:120. doi: 10.1186/s13034-023-00670-3 (PMC10583422; doi:10.1186/s13034-023-00670-3)
Supplement: Supplementary file 2 — Additional file 2: Table S2. Bivariate correlations of all examined variables [file 13034_2023_670_MOESM2_ESM.docx]

**Table S2.** *Bivariate correlations of all examined variables*

| Variables | *M* | *SD* | *N* | 1 | 2 | 3 | 4 | 5 | 6 | 7 | 8 | 9 | 10 | 11 | 12 | 13 | 14 | 15 | 16 | 17 |
| --- | --- | --- | --- | --- | --- | --- | --- | --- | --- | --- | --- | --- | --- | --- | --- | --- | --- | --- | --- | --- |
| *SR facets* |  |  |  |  |  |  |  |  |  |  |  |  |  |  |  |  |  |  |  |  |
| 1. Working-memory updating | 6.22 | 1.47 | 1435 | - | - | - | - | - | - | - | - | - | - | - | - | - | - | - | - | - |
| 2. Cognitive flexibility/set-shifting | 15.71 | 4.59 | 1437 | .352** | - | - | - | - | - | - | - | - | - | - | - | - | - | - | - | - |
| 3. Inhibition^1^ | 24.91 | 8.80 | 1437 | -.265** | -.327** | - | - | - | - | - | - | - | - | - | - | - | - | - | - | - |
| 4. Inhibitory control | 3.54 | 0.67 | 1303 | .103** | .142** | -.113** | - | - | - | - | - | - | - | - | - | - | - | - | - | - |
| 5. Planning behavior | 3.76 | 0.88 | 1197 | .266** | .290** | -.297** | .296** | - | - | - | - | - | - | - | - | - | - | - | - | - |
| 6. Affective decision-making | 5.41 | 11.35 | 1437 | .069** | .093** | -.030 | .015 | .022 | - | - | - | - | - | - | - | - | - | - | - | - |
| 7. Delay of gratification | 2.80 | 1.23 | 1356 | .052 | .093** | -.060* | .032 | .048 | .028 | - | - | - | - | - | - | - | - | - | - | - |
| 8. Emotional reactivity | 2.20 | 0.71 | 1286 | -.096** | -.055* | .096** | -.401** | -.164** | -.038 | -.056 | - | - | - | - | - | - | - | - | - | - |
| 9. Anger regulation | 2.19 | 0.66 | 1294 | -.009 | -.013 | -.023 | .133** | -.012 | -.031 | -.014 | -.329** | - | - | - | - | - | - | - | - | - |
| 10. Heart-rate variability | 58.89 | 27.99 | 1283 | -.001 | .004 | .072* | -.046 | -.033 | .047 | .069* | .013 | -.038 | - | - | - | - | - | - | - | - |
| *Risk factors* |  |  |  |  |  |  |  |  |  |  |  |  |  |  |  |  |  |  |  |  |
| 11. Gender^2^ | - | - | 1453 | .004 | -.159** | .080** | -.111** | -.189** | .083* | .078* | -.012 | -.050* | .090** | - | - | ´- | - | - | - | - |
| 12. Education status | 5.05 | 1.01 | 1325 | .189** | .177** | -.087* | .168** | .261** | .067* | .032 | -.029 | -.106** | -.008 | .012 | - | - | - | - | - | - |
| 13. Family adversity | 0.95 | 1.29 | 1135 | -.047 | -.080** | .049^+^ | -.146** | -.164** | -.066* | -.029 | .170** | -.015 | -.014 | -.046 | -.194** | - | - | - | - | - |
| 14. Peer problems | 0.22 | 0.26 | 1008 | -.094* | -.103** | .106** | -.281** | -.422** | - .025 | .001 | .290** | -.049 | -.020 | .063* | -.117** | .156** | - | - | - | - |
| *Internalizing symptoms (INT)* |  |  |  |  |  |  |  |  |  |  |  |  |  |  |  |  |  |  |  |  |
| 15. INT (t1) | 1.70 | 1.79 | 1314 | -.098** | -.002 | .050 | -.134** | -.196** | -.033 | -.027 | .417** | -.091* | -.022 | -.061 | -.126** | .229** | .253** | - | - | - |
| 16. INT (t2) | 1.65 | 1.81 | 1173 | -.120** | -.048 | .056 | -.142** | -.165** | -.044 | -.070* | .345** | -.060^+^ | -.031 | -.075** | -.129** | .258** | .246** | .631** | - | - |
| 17. INT (t3) | 1.61 | 1.86 | 1047 | -.110** | -.027 | .053 | -.192** | -.225** | -.045 | -.077* | .323** | -.069* | -.030 | -.066* | -.157** | .199** | .251** | .533** | .560** | - |
| ^1^non-inverted interference score of the fruit stroop: higher values indicate lower inhibition capability  ^2^Spearman’s Rho was used for gender (0 = female, 1 = male)  ^+^*p* < .10, **p* < .05, ***p* < .001 | | | | | | | | | | | | | | | | | | | | |
