# Supplementary material for: Self-regulation facets differentially predict internalizing symptom trajectories from middle childhood to early adolescence: a longitudinal multimethod study
Source: Child Adolesc Psychiatry Ment Health. 2023 Oct 17;17:120. doi: 10.1186/s13034-023-00670-3 (PMC10583422; doi:10.1186/s13034-023-00670-3)
Supplement: Supplementary file 3 — Additional file 3: Table S3. Descriptive data on SR facets and risk factors for the latent trajectory classes [file 13034_2023_670_MOESM3_ESM.docx]

**Table S3.** *Descriptive data for latent trajectory classes in SR facets and risk factors (raw scores)*

| Variables | Total | | Class 1  stable low | | Class 2  increasing | | Class 3  decreasing | |
| --- | --- | --- | --- | --- | --- | --- | --- | --- |
|  | *M* | (*SD*) | *M* | (*SD*) | *M* | (*SD*) | *M* | (*SD*) |
| *SR facets* |  |  |  |  |  |  |  |  |
| Working-memory updating | 6.22 | (1.47) | 6.30 | (1.46) | 5.91 | (1.47) | 5.80 | (1.44) |
| Cognitive flexibility/set-shifting | 15.71 | (4.59) | 15.77 | (4.52) | 15.66 | (4.65) | 15.22 | (5.11) |
| Inhibition^1^ | 24.91 | (8.80) | 24.74 | (8.61) | 25.66 | (10.71) | 25.79 | (8.46) |
| Inhibitory control | 3.53 | (0.67) | 3.33 | (0.71) | 3.34 | (0.67) | 3.57 | (0.65) |
| Planning behavior | 3.76 | (0.88) | 3.83 | (0.86) | 3.43 | (0.87) | 3.41 | (0.95) |
| Affective decision-making | 5.41 | (11.35) | 5.52 | (11.32) | 3.63 | (9.56) | 6.20 | (13.09) |
| Delay of gratification | 2.80 | (1.23) | 2.85 | (1.20) | 2.63 | (1.37) | 2.55 | (1.24) |
| Emotional reactivity | 2.20 | (0.71) | 2.09 | (0.66) | 2.68 | (0.77) | 2.82 | (0.71) |
| Anger regulation | 2.19 | (0.66) | 2.21 | (0.63) | 2.04 | (0.78) | 2.08 | (0.70) |
| Heart-rate variability | 58.89 | (27.99) | 59.07 | (28.26) | 56.65 | (26.21) | 59.51 | (27.22) |
| *Risk factors^2^* |  |  |  |  |  |  |  |  |
| Education status | 5.05 | (1.01) | 5.10 | (0.99) | 4.83 | (0.99) | 4.77 | (1.13) |
| Family adversity | 0.95 | (1.29) | 0.84 | (1.19) | 1.65 | (1.62) | 1.39 | (1.58) |
| Peer problems | 0.22 | (0.26) | 0.20 | (0.25) | 0.34 | (0.29) | 0.36 | (0.33) |
| ^1^non-inverted interference score of the fruit stroop: higher values indicate lower inhibition capability  ^2^Gender distributions in trajectory classes are described in the manuscript | | | | | | | | |
